# Supportive care: Comparing exercise interventions for upper extremity polyneuropathy induced by chemo- or immunotherapy — VISCIPH B

**DOI:** 10.1007/s00520-026-10459-7

**Published:** 2026-03-19

**Authors:** Stefanie Siebert, Jane Kersten, Sarah Man, Sarina Heinz, Katharina Leuchte, Freerk T. Baumann, Timo Sonntag

**Affiliations:** 1 Department I of Internal Medicine, Center for Integrated Oncology Aachen Bonn Cologne Duesseldorf, University Hospital of Cologne, Kerpener Str. 62, Cologne, 50937 Germany; 2 University of Cologne, Cologne, Germany; 3 Department of Oncology, National Center for Cancer Immune Therapy (CCIT-DK), Copenhagen University Hospital Herlev, Herlev, Denmark

**Keywords:** Therapy-induced peripheral neuropathy, Upper extremities, Sensorimotor exercise, Vibration intervention, Cancer treatment toxicity, Supportive care

## Abstract

**Background:**

Chemotherapy and immunotherapy-induced peripheral neuropathy affects up to 68% of cancer patients and may persist long after treatment, substantially impairing daily functioning and quality of life. While exercise therapy has demonstrated benefits in lower-limb polyneuropathy (PNP), evidence for upper-extremity symptoms remains scarce. The VISCIPH B pilot study investigated the effect of two supervised exercise interventions for PNP of the upper extremities using exploratory analyses of symptom response.

**Methods:**

In this single-center randomized controlled pilot trial (DRKS00023287), 61 cancer patients with symptomatic upper-extremity PNP were randomized (1:1) to either (a) combined sensorimotor plus vibration exercise (PNPEX) or (b) moderate resistance exercise (MREX). Both interventions were carried out supervised twice weekly over 12 weeks. Feasibility outcomes included adherence, retention, assessment completeness, and safety of the exercise interventions. Symptom outcomes were assessed with the FACT/GOG-Ntx questionnaire, measures of pain (NRS), depth sensitivity (Rydel-Seiffer tuning fork), and quality of life (EORTC QLQ-C30).

**Results:**

Feasibility criteria showed high adherence (86%) and retention (69%) rates. A total of 42 patients (mean age 53.3 years, 36% male) completed the intervention with no reported intervention-related adverse events. In the exploratory effect analyses, 50% of PNPEX participants (10/20) were classified as responders, compared to 14% (3/21) in MREX (OR = 5.45, *p* = 0.043). FACT/GOG-Ntx scores improved significantly in PNPEX (*p* = 0.017) but not in MREX (*p* = 0.46), resulting in a significant difference between the two groups (*p* = 0.05). Patient-reported outcomes revealed significant improvements in the PNPEX group regarding numbness and tingling (NRS), depth sensitivity at four of the eight tested bone sites and global health (*p* = 0.001).

**Conclusion:**

The VISCIPH B pilot trial confirmed the feasibility, safety, and acceptance of supervised exercise for upper-extremity PNP in cancer patients. Significant improvements in patient-reported and functional outcomes indicate that combined sensorimotor and vibration exercise can meaningfully reduce PNP symptoms and should be evaluated in larger trials.

**Supplementary information:**

The online version contains supplementary material available at 10.1007/s00520-026-10459-7.

## Introduction

Polyneuropathy (PNP) is one of the most common and distressing side effects of modern cancer therapy, including neurotoxic chemotherapy (e.g., platinum compounds, taxanes, vinca alkaloids, proteasome inhibitors) or selected immunotherapy-related agents. It affects up to 68% of patients within the first month after treatment, with symptoms persisting in 30% of patients for 6 months or longer [1, 2]. PNP can severely impair quality of life (QoL), manual dexterity, and physical function. Symptoms can include numbness, tingling, pain and motor impairment, and often affect the distal extremities symmetrically. Although research has primarily focused on the lower extremities, PNP in the upper limbs can also cause significant functional limitations, particularly in occupation-related tasks and activities of daily living [3–5].

Current clinical practice guidelines indicate limited pharmacological options for chemotherapy-induced peripheral neuropathy (CIPN). While duloxetine has shown moderate efficacy for established painful CIPN, no pharmacological treatment has demonstrated consistent benefits across the full spectrum of sensory and functional PNP symptoms, particularly non-painful manifestations [6, 7]. Exercise therapy has therefore emerged as a promising non-pharmacological strategy. Clinical studies indicate that sensorimotor, vibration, resistance-based, and aerobic exercise interventions may alleviate sensory and motor symptoms of PNP — predominantly shown in the lower extremities so far [8–10]. These modalities are assumed to enhance neuromuscular control, proprioceptive input, and pain modulation [11, 12]. Similar neurophysiological mechanisms are assumed to apply to the upper extremities, although direct evidence remains limited. Nevertheless, the most effective exercise modality remains unclear and requires further investigation to guide targeted and efficient therapeutic regimens.

Evidence on exercise-based therapy for PNP in the upper extremities remains scarce. Based on these findings, it is of great interest to evaluate the comparative efficacy of different exercise interventions as possible treatment options for PNP in cancer patients. Previous pilot studies on exercise therapy for upper extremity PNP have reported beneficial effects of sensorimotor, vibration-based, and resistance exercise on sensory function, hand grip strength, vibration sensitivity, and symptom burden, suggesting potential clinical benefits of targeted exercise interventions for this patient group [3–5, 8, 13]. However, these studies were limited by small sample sizes and substantial inter-individual variability in treatment response, with analyses largely restricted to group-level effects. As a result, it remains unclear which patients benefit most from specific exercise modalities, highlighting the need for responder-focused analyses.

It is increasingly recognized that patients respond differently to exercise interventions. Various factors have been suggested to influence individual treatment response, including baseline neuropathy severity, type and cumulative dose of neurotoxic therapy, physical activity levels, and neuromuscular capacity. A randomized clinical trial by Streckmann et al. (2024) demonstrated that neuromuscular training can substantially reduce the incidence of chemotherapy-induced neuropathy. The trial also highlighted pronounced inter-individual variability in treatment effects [13]. Distinguishing responders from non-responders is an important step towards optimizing supportive care strategies [13, 14].

The VISCIPH B study compares the effects of two supervised exercise interventions on neuropathy-related symptoms of the upper extremities in patients undergoing chemo- or immunotherapy: combined upper-extremity vibration and whole-body sensorimotor exercise (PNPEX) versus moderate full-body resistance exercise (MREX). Within this randomized pilot trial, the study primarily explores differences in neuropathy-related outcomes between the two interventions, while feasibility parameters are reported descriptively. Exploratory responder analyses were conducted to generate hypotheses for future confirmatory trials.

## Methods

### *Design and participants*

This randomized pilot trial (DRKS-ID: DRKS00023287; registered October 15, 2020) was conducted as a prospective single-center study. Approval was granted by the Ethics Committee of the Medical Faculty at the University of Cologne (reference number 17–165). The aim of the VISCIPH B trial was to compare the effects of both exercise interventions on neuropathy-related outcomes. Secondly, responder analysis. All neuropathy-related outcomes presented in this manuscript represent secondary, exploratory analyses.

The study was conducted at the Center for Integrated Oncology (CIO) at Cologne University Hospital. Between September 2020 and February 2022, adult patients (≥ 18 years) with a confirmed cancer diagnosis who were currently receiving neurotoxic systemic cancer therapy were evaluated for inclusion in the study. To be eligible for the study, participants had to exhibit clinically relevant sensory symptoms in their upper extremities at the start, such as numbness, tingling, or burning sensations. Patients were permitted to enroll if they had previously received neurotoxic therapy unless an alternative cause of polyneuropathy was present. Medical clearance by the treating physician and written informed consent of the patient were mandatory for study inclusion.

Neurotoxic agents were defined as taxanes, platinum-based compounds, vinca alkaloids, epothilones, bortezomib, and immune checkpoint inhibitors (e.g., nivolumab, pembrolizumab). To reduce confounding variables, individuals with pre-existing polyneuropathy from comorbidities such as diabetes mellitus or chronic alcohol abuse were excluded. Additional exclusion criteria were medical contraindications to physical exercise — especially vibration exercises — including osteolysis, unstable bone metastases, recent thrombosis, cervical disc prostheses, or joint arthroplasties.

**Figure 1:**
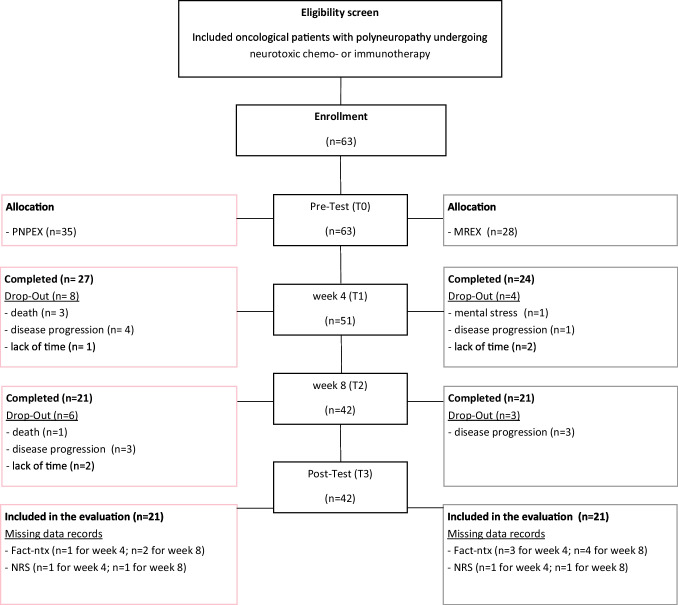
Flowchart VISCIPH B

Eligible participants were randomly assigned in a 1:1 ratio to one of two intervention groups using a computer-generated block randomization (patient flow in Fig. 1). The assignment was kept secret from both the participants and the research staff during the baseline examinations and was only revealed after their completion. The two intervention arms consisted of:

- Combining upper-extremity vibration and sensorimotor exercise (PNPEX)
- Moderate full-body resistance exercise (MREX)

### *Exercise intervention*

Both groups participated in structured and protocolized exercise interventions twice a week for 12 weeks. Each 60-min session was supervised by a qualified sports scientist at Cologne University Hospital’s Oncological Training Therapy (OTT) facility. Each session began with a 10-min, moderate-intensity warm-up using Technogym equipment (such as a bicycle ergometer, treadmill, or elliptical walker) at a Rate of Perceived Exertion (RPE) of 5–6. This was followed by 30–45 min of group-specific exercise and concluded with a 10-min cool-down at an RPE of 5–6. Participants who attended fewer than 50% of the sessions were classified as non-completers.

### *Vibration and sensorimotor exercise (PNPEX)*

The PNPEX group performed combined vibration (side-alternating plate, Galileo Fit, Novotec Medical) and sensorimotor exercises for the upper extremities. Each session included four vibration rounds per training unit. The vibration started at 18 Hz for 30 s, increasing by 1 Hz per exercise session up to 36 Hz. From the 19th session onward, the protocol was repeated at 18 Hz for 60 s, with rest periods equal to or longer than exercise time. A standardized posture was maintained (see supplements), and modifications were permitted if needed. Sensorimotor exercise progressed from bipedal to monopedal tasks and from static to dynamic exercises, based on a predefined catalogue. Further, Therabands® resistance bands and balance boards were used to enhance proprioception and neuromuscular challenges and achieve the target level of difficulty (RPE 5–6). The supplementary files contain a comprehensive description of the intervention.

### *Moderate resistance exercise (MREX)*

The MREX group performed full-body moderate resistance training two sets of 20 repetitions at 50–60% 1RM (RPE 5–6) on six strength machines covering the major muscles (total abdominal, low row, chest press, lower back, leg curl, and leg press), with 60-s rest periods between sets.

### *Measurements*

Quantitative sensory testing was carried out before and after the 12-week intervention. In addition to the baseline (T0) and post-intervention (T3) assessments, paper-based questionnaires were administered on site at weeks 4 (T1), 8 (T2), and 12 (T3) of the intervention period in order to capture subjective symptoms of peripheral neuropathy (PNP).

### *Responder/non-responder-analyses*

The FACT/GOG-Ntx questionnaire was chosen because it is a validated, widely used instrument designed specifically to assess chemotherapy-induced PNP and its impact on the abilities of cancer patients [15, 16]. Covering sensory, motor, and functional domains, it is well-suited to identify clinically relevant changes in neuropathy-related symptoms and their impact on daily life.

To classify participants as either responders or non-responders of the intervention, the standard deviation (SD) of the change in scores from baseline (T0) to the post-intervention stage (T3) was calculated. The SD was 8.02 points. Following a distribution-based approach, this value (4.01 points) was divided by 2 and used as the minimal important difference (MID) threshold. Participants who improved by more than 4 points on the FACT/GOG-Ntx total score were classified as responders, while the others were classified as non-responders. Previous research has demonstrated the consistent applicability of the half SD threshold as an indicator of clinically meaningful change across health-related QoL measures [17].

### *Patient-reported outcomes (PROs)*

The following assessments were additionally used to determine the incidence and severity of PNP:

- Peripheral depth sensitivity was assessed with a Rydel-Seiffer tuning fork (128 Hz) on a graded scale from 0 (no sensitivity) to 8 (highest sensitivity), representing a form of quantitative sensory testing [18]. The measurement was taken on both upper extremities at the following measuring sites: ulnar styloid process and I, III, and V carpometacarpal joint).
- Health-related quality of life was assessed using the EORTC QLQ-C30, a 30-item self-report questionnaire covering physical, emotional, cognitive, and social functioning as well as symptom scales, scored according to standard EORTC guidelines (0–100; higher scores indicate better functioning or greater symptom burden, depending on the scale) [19].
- Neuropathic pain as well as numbness and tingling in the upper extremity was assessed using an 11-point Numeric Rating Scale (NRS; 0 = no symptom, 10 = worst imaginable symptom). The NRS is a valid and reliable instrument for assessing neuropathic symptoms with high sensitivity to change [20].

### *Statistical analysis*

All analyses were conducted using SPSS Statistics version 26 (IBM). A per-protocol analysis was performed. Given the pilot nature of the study, no formal power calculation was conducted. Responder status was defined a priori as an improvement of more than 4 points (half of the standard deviation) in the FACT/GOG-Ntx total score from baseline to post-intervention [17]. This responder classification was used for exploratory analyses only. Pre–post changes and group differences were analyzed using mixed-design ANOVAs with repeated measures. Fisher’s exact test was used for responder analyses. Due to limited sample size, no confounding variables were included as covariates; potential confounders were examined descriptively. A two-sided significance level of *p* < 0.05 was applied. Missing data were evaluated and handled based on their extent and pattern. Figure 1 illustrates the study flow and missing data.

## Results

### *Study population and characteristics*

A total of 61 cancer patients undergoing active treatment were enrolled in the VISCIPH B study. Nineteen patients (31%) discontinued the intervention due to disease progression (*n* = 9) and death (*n* = 5, Fig. 1). Forty-two patients completed the intervention and were included in the per-protocol analyses. Twenty-seven patients (64.3%) were female, with a mean age of 53.3 years (range 41–63 years). Half of the participants presented with metastatic disease at baseline.

The most common diagnosis was breast cancer (*n* = 12; 28.6%), followed by multiple myeloma (*n* = 6; 14.3%) and lymphatic malignancies (*n* = 4; 7.1%). Other tumor entities included pancreatic, lung, and prostate cancer (Table 1). In terms of cancer therapy, 26 patients (61.9%) received platinum-based agents or taxanes, ten patients (23.8%) underwent immunotherapy, and the remaining six patients (14.3%) received vinca alkaloids. Baseline characteristics and treatment protocols showed no significant differences between both groups.

**Table 1:** Baseline demographics

	Patients (*n* = 42)						
	PNPEX (*n* = 21)			MREX (*n* = 21)			
Female	14 (66.67%)			13 (61.90%)			
Male	7 (33.33%)			8 (38.10%)			
	*M* ± SD	Min	Max	*M* ± SD	Min	Max	*p*-value
Antropometric data							
Age (y)	53.29 ± 6.27	41	63	52.19 ± 6.28	40	62	0.602
BMI (kg/m^2^)	26.10 ± 6.46	18.47	40.48	24.14 ± 4.80	17.08	38.27	0.271
Disease characteristics							
Cancer sites							
Breast			7 (33%)			5 (24%)	
Multiple myeloma			3 (14%)			3 (14%)	
Pancreas			2 (9%)			1 (5%)	
Lymphoma			2 (9%)			2 (9%)	
Lung			1 (5%)			0 (0%)	
Gynecological			2 (9%)			1 (5%)	
Head and neck			2 (9%)			1 (5%)	
Prostate			0 (0%)			1 (5%)	
Gastrointestinal			1 (5%)			7 (33%)	
Soft tissue sarcomas			1 (5%)			0 (0%)	
Metastatic disease							
Liver			4 (19%)			4 (19%)	
Lungs			4 (19%)			4 (19%)	
Bones			2 (10%)			2 (10%)	
Peritoneum			1 (5%)			0 (0%)	
Lymph nodes			1 (5%)			3 (14%)	
Neurotoxic therapy type							
Only taxanes			5 (24%)			5 (24%)	
Only platinums			4 (19%)			8 (38%)	
Only vinca alkaloids			4 (19%)			2 (10%)	
Taxanes and platinums			2 (10%)			2 (10%)	
Inhibitors			4 (19%)			1 (5%)	
Immunochemotherapy			2 (10%)			3 (14%)	

### *Secondary symptom-specific outcome*

**Figure 2:**
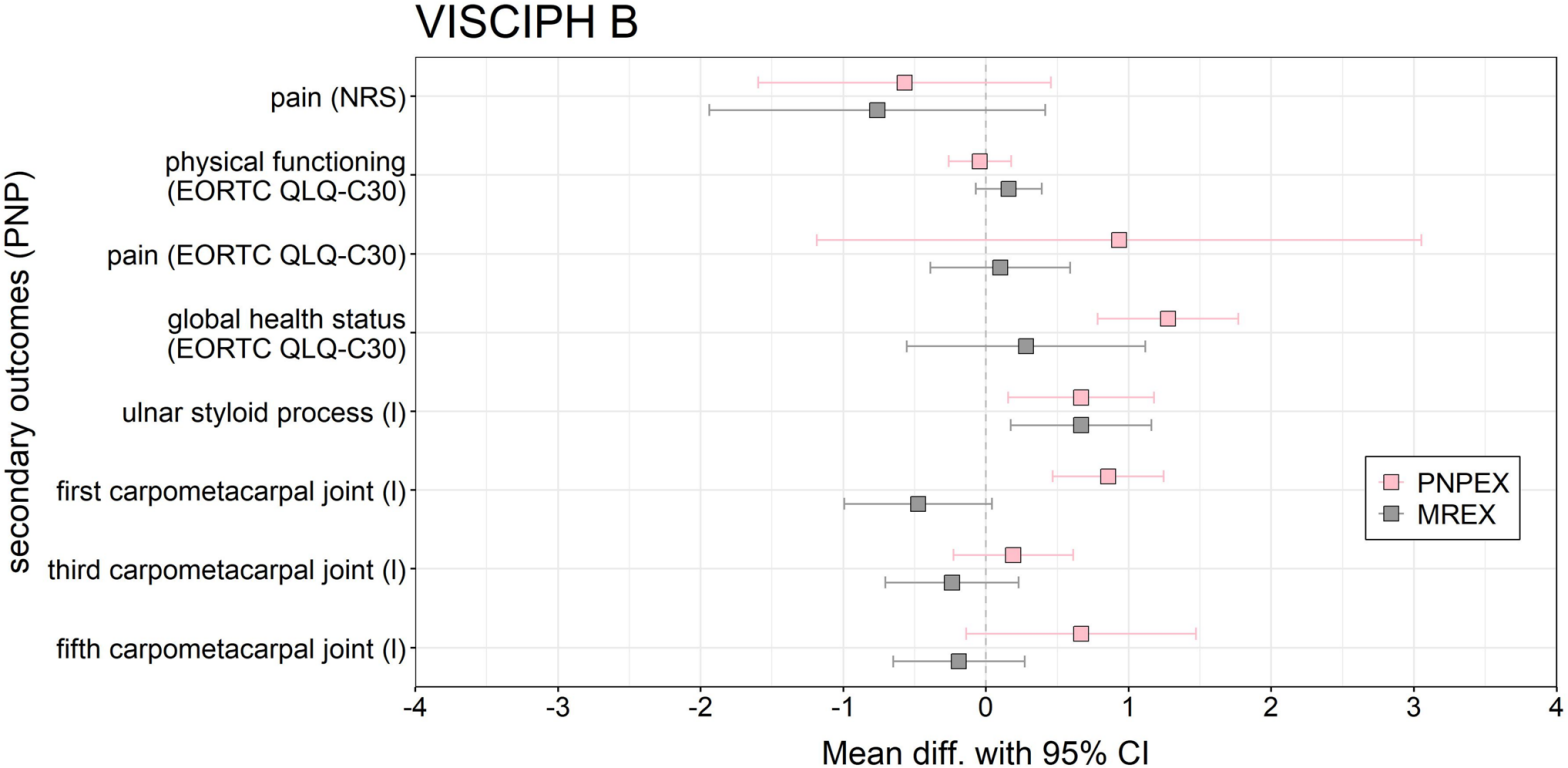
Forest plot of the effects of PNPEX vs. MREX on secondary outcomes on PNP for VISCIPH B. Data are reported as mean difference (pre–post-comparison) (95% CI)

The following section presents the symptom-specific outcomes of the 42 patients who completed the intervention (Fig. 2). Regarding the pain outcome (NRS), both intervention groups demonstrated a slight reduction in reported pain intensity after the 12-week intervention period. The MREX group showed a mean decrease of −0.76 (95% CI, −1.94 to 0.42; *p* = 0.060) while the PNPEX group reported a mean reduction of −0.57 (95% CI, −1.59 to 0.45; *p* = 0.594). Regarding EORTC physical functioning, neither intervention resulted in statistically significant changes over the intervention period. Both groups experienced an improvement in global health status, with a greater mean increase observed in the PNPEX group (+ 1.28; 95% CI, 0.78–1.77; *p* = 0.001) than in the MREX group (+ 0.28; 95% CI, −0.55 to 1.12; *p* = 0.518). Regarding the EORTC QLQ-C30 pain scale, both groups showed small, non-significant changes over the intervention period.

Both groups showed a significant improvement in depth sensitivity of 0.67 (*p* = 0.018) at the left styloid process of the ulna. Additionally, the PNPEX group demonstrated a notable improvement of + 0.86 (95% CI, 0.47–1.25) at the first carpometacarpal joint (*p* = 0.002). By contrast, the MREX group showed a slight deterioration (−0.48; 95% CI, −0.99 to 0.04; *p* = 0.049), which should be interpreted cautiously given the exploratory nature of the analyses.

### *Responder/non-responder analyses (FACT/GOG-Ntx)*

Applying the MID threshold of FACT/GOG-Ntx questionnaire, patients were classified into responders and non-responders.

This resulted in a significant odds ratio of 5.45 (*p* = 0.043; *Fisher’s exact test*), indicating that participants in the PNPEX group were *more likely to be classified as responders* compared to the MREX group (Table 2).

**Table 2:** Distribution of responders and non-responders by intervention group (FACT/GOG-Ntx)

	MREX	PNPEX
Responder	3	10
Non-responder	18	11

The pre–post analyses demonstrated a significant reduction in mean FACT/GOG-Ntx scores in the PNPEX group (*p* = 0.017), whereas no significant change was observed in the MREX group (*p* = 0.46). Assuming sphericity, a statistically significant time × group interaction was observed (*F*(3.84) = 2.67; *p* = 0.05), suggesting differential symptom trajectories between the two intervention groups.

### *Responder/non-responder — PNPEX*

A total of 21 patients from the PNPEX group were included in the responder analysis. Based on predefined criteria, ten participants (47.6%) were classified as responders and 11 as non-responders. There were no significant differences between both groups in terms of sex distribution, age or body mass index (BMI). The mean age was 52.1 ± 7.0 years for responders and 52.3 ± 5.9 years for non-responders (*t*-test, *p* = 0.952). The mean BMI was 26.0 kg/m^2^ for responders and 25.1 kg/m^2^ for non-responders (Mann–Whitney *U* test, *p* = 0.918). There was no significant difference in sex distribution between the groups (Fisher’s exact test, *p* = 1.000; OR = 0.76), or in the presence of metastases (OR = 0.84, *p* = 1.000), chemotherapy (OR = 0.52, *p* = 1.000) or immunotherapy (OR = 1.47, *p* = 1.000).

Baseline handgrip strength (T0) showed lower mean values in responders, but these differences were not statistically significant: right hand: 28.5 ± 12.3 kg vs. 37.0 ± 16.0 kg, *p* = 0.185; left hand: 27.3 ± 11.2 kg vs. 33.2 ± 16.1 kg, *p* = 0.342.

There were also no significant differences in training adherence or intervention length. Mean adherence was 88.7% in responders and 89.5% in non-responders (*p* = 0.773). The mean intervention period was 10.4 weeks versus 12.2 weeks (*p* = 0.859). Pre-existing physical activity did not appear to be associated with responder status. Seven of ten responders and eight of ten non-responders reported a history of regular sports participation (Fisher’s exact test, *p* = 1.000; OR = 1.14). In conclusion, no statistically significant predictors for responder status could be identified across all analyzed variables — sociodemographic, clinical, functional, and behavioral (all *p* ≥ 0.185).

## Discussion

The aim of this study was to evaluate the potential effects of two targeted exercise interventions on symptoms of polyneuropathy in the upper extremities of cancer patients. Both the sensorimotor/vibration exercises (PNPEX) and the moderate resistance exercises (MREX) proved to be acceptable and safe to perform. However, PNPEX showed more significant improvements in selected neuropathy-related outcomes such as the FACT/GOG-Ntx or numbness/tingling (see supplements) [5]. PNPEX not only led to positive developments in terms of vibration sensitivity and symptom reduction, but also to improvements in depth sensitivity and paresthesia at four of eight measurement points (see supplements). This suggests that sensorimotor/vibration-based movement therapy is both feasible and clinically relevant for therapy-related neuropathic symptoms in the upper extremity.

Analysis of response probabilities based on the FACT/GOG-Ntx demonstrated substantial differences between the groups. The results suggest that PNPEX may offer more targeted benefits for PNP symptoms than resistance exercise alone. The FACT/GOG-Ntx, which was originally developed and validated by Cella et al. in 1993 [15], is particularly well-suited to detect subtle changes in neuropathy-related symptoms, and has been established as a sensitive endpoint in PNP research [4]. The findings are consistent with previous studies indicating that sensorimotor and vibration exercise can enhance proprioception and vibration sensitivity, thereby alleviating neuropathic symptoms [8, 9, 13, 14, 21]. Recent results from the VISCIPH A trial further support this interpretation, as PNPEX effectively prevented the deterioration of deep sensitivity in the upper extremities and demonstrated favorable effects on overall health compared to resistance exercise [5]. While resistance intervention remains important for improving strength and physical functioning in cancer survivors, its specific impact on neuropathic symptoms appears to be less pronounced.

Within the PNPEX group, no sociodemographic, clinical, functional, or behavioral variables predicted responder status, reflecting the variability in the response to PNP exercise reported previously [8, 14]. The findings of this randomized controlled trial further support the idea that sensorimotor intervention has high neuro-modulating potential, stimulating the regenerative and adaptive mechanisms of muscle spindles and sensory afferent nerves to promote neural plasticity [13]. In contrast, vibration exercise appears to primarily activate more superficial nerves [13]. These observations suggest that, when regularly trained under progressive load, the human neuromuscular system may preserve neural functions even during chemotherapy. This is consistent with preclinical models showing the potential for peripheral nerve regeneration through activity-dependent mechanisms. In this context, Kleckner et al. (2018) demonstrated that exercise may also modulate central neural processing in cancer survivors, suggesting a dual peripheral–central mechanism [11].

Analysis of the secondary outcomes revealed mainly small changes, with relevant group-specific differences. Both groups showed a reduction in symptoms on the NRS pain scale, although these changes did not reach statistical significance. Conversely, we observed an increasing trend in pain scores on the EORTC QLQ-C30 scale, particularly in the PNPEX group. The divergence between the two pain measures may be explained by their different conceptual focus: the NRS specifically assesses neuropathic pain in the hands, whereas the EORTC QLQ-C30 captures general pain, including tumor- and therapy-related joint or musculoskeletal pain that may accumulate during treatment [4]. Self-reported physical function remained largely unchanged, with a small improvement in the MREX group and minimal changes in the PNPEX group. Comparable intervention studies in patients with chemotherapy-induced neuropathy have also reported only modest changes in physical function [8, 14, 22], suggesting that these domains may require longer or multimodal exercise interventions to achieve measurable benefits. Furthermore, the post-intervention pain scores of both groups fell within the range reported for the general cancer survivor population [23, 24]. This suggests that exercise interventions may primarily stabilize, rather than substantially improve pain perception in the short term. However, global health status improved significantly in the PNPEX group, while the MREX group showed only a small, non-significant increase. This finding may suggest that reductions in neuropathic symptoms can translate into meaningful improvements in overall QoL, as reported in previous research linking symptom burden and health-related QoL in cancer survivors [24, 25].

In terms of depth sensitivity, both groups improved at the ulnar styloid, but only the PNPEX group showed a significant gain at the first carpometacarpal joint. At the third and fifth carpometacarpal joints, PNPEX again showed more favorable trajectories than MREX, though without statistical significance. These findings are consistent with previous studies indicating that sensorimotor and vibration exercise can improve proprioception and preserve sensory function in patients with chemotherapy-induced peripheral neuropathy [8, 13, 14, 21], whereas resistance exercise appears to be less effective in this area. Alongside the higher response rate in the PNPEX group, these results highlight the potential clinical relevance of neuromuscular exercise modalities. This supports earlier observations that neuromuscular exercise interventions can slow down PNP progression and maintain physical function [22, 25].

However, these findings should be interpreted with caution. Although depth sensitivity values below 5 mm for individuals under 60 years old and below 4 mm for individuals aged 60 years and over are classified as pathological, mean values in our cohort (5.94–7.18 mm) were consistently above these thresholds despite acute neuropathy [26]. This calls into question the clinical relevance of the observed changes and highlights the limitations of the tuning fork test, which is highly dependent on the examiner. More objective methods, such as multi-frequency vibrometry or biothesiometry, could prove to be more precise [27]. Early pilot data also suggest that standardized vibratory approaches could improve measurement accuracy and have therapeutic potential [28].

These findings are supported by previous publications demonstrating beneficial effects of sensorimotor and vibration-based exercise on upper-extremity sensory function and patient-reported outcomes [8, 13, 14, 21]. The above findings emphasize the need for future interventional studies for both extremities to improve understanding of the impact of therapy-related PNP on patients’ daily lives. From a clinical perspective, the timing of such interventions also appears to be important: exercise may be beneficial during active therapy when neuropathic damage occurs, as well as during the rehabilitation phase. Early, targeted exercise may help slow down the progression of symptoms or support partial recovery [5], which highlights the importance of integrating structured physical interventions into cancer treatment.

### *Limitations*

Several limitations should be taken into account when interpreting these results. Firstly, the relatively small sample size reduced statistical power and may limit the generalizability of the findings. Secondly, information on previous therapies was incomplete for some patients, meaning potential influencing factors could not be controlled in all cases. Thirdly, drug dosage was not fully standardized or consistently documented, so some degree of variability in treatment exposure and potential influence on the results cannot be ruled out. Additionally, the relatively short intervention and observation periods, which were partially non-overlapping, limited the ability to assess the potential long-term effects of the drug therapy [25]. Consequently, potentially relevant developments outside the study window were not considered. Most dropouts were related to disease progression or death rather than intervention-related factors; however, the absence of a formal comparison between completers and non-completers limits conclusions regarding potential attrition bias. The primary responder analysis used the FACT/GOG-Ntx, which assesses CIPN in both the upper and lower extremities. While this captures relevant functional impairment, it does not exclusively measure symptoms in the upper extremities. Finally, the patient recruitment, program implementation, and data collection were complicated by the COVID-19 pandemic restrictions, which did not lead to critical protocol deviations that would substantially bias the interpretation of the results.

## Conclusion

Our findings indicate that the sensorimotor/vibration program was well accepted and safely implemented and was associated with improvements in neuropathy-related symptoms, whereas the muscle resistance program (MREX), while similarly acceptable, showed no such improvements [5]. The observation that the PNPEX group exhibited more favorable outcomes suggests that targeted neuromuscular exercise modalities may offer additional clinical benefits. The responder/non-responder analysis further highlights substantial inter-individual variability, indicating that individual responses to exercise-based interventions vary considerably and cannot yet be reliably predicted. While the current ASCO guidelines recommend dose modification of duloxetine as the only evidence-based option for symptom relief [24], our results suggest that specific exercise modalities, particularly sensorimotor and vibration training, may represent a promising adjunctive approach to reduce the severity of neuropathic symptoms in oncology patients. Alongside previous findings from VISCIPH A, which showed benefits when exercise was started early in therapy, these results highlight the potential value of initiating targeted interventions as soon as neuropathic symptoms appear. Exercise plays a pivotal role in maintaining and even restoring neuromuscular function, and should be considered as part of a multimodal PNP management strategy, pending confirmation in larger controlled trials.

## Supplementary information

Below is the link to the electronic supplementary material.

ESM 1 (PDF 169 KB)

ESM 2 (PDF 241 KB)

## Data Availability

Data are available upon reasonable request.
