# Supplementary material for: Supportive care: Comparing exercise interventions for upper extremity polyneuropathy induced by chemo- or immunotherapy — VISCIPH B
Source: Support Care Cancer. 2026 Mar 19;34(4):345. doi: 10.1007/s00520-026-10459-7 (PMC13002742; doi:10.1007/s00520-026-10459-7)
Supplement: Supplementary file 1 — (PDF 169 KB) [file 520_2026_10459_MOESM1_ESM.pdf]

**VISCIPH B**

| ***PERFORMANCE*** | | PNPEX (n = 21) | | | MREX (n = 21) | | |
|---|---|---|---|---|---|---|---|
| | | Baseline | Post-Test | | Baseline | Post-Test | |
| *Depth sensitivity* | | M ± SD | M ± SD | p-value | M ± SD | M ± SD | p-value |
| *ulnar styloid process* | *right* | 6.00 ± 0.95 | 6.62 ± 0.74 | **0.016*** | 6.24 ± 1.14 | 6.05 ± 1.72 | 0.775 |
| | *left* | 6.10 ± 1.10 | 6.76 ± 0.77 | **0.018*** | 6.00 ± 1.00 | 6.67 ± 1.24 | **0.018*** |
| *I carpometacarpal joint* | *right* | 6.86 ± 0.79 | 7.19 ± 0.81 | 0.117 | 7.05 ± 0.92 | 6.86 ± 1.06 | 0.449 |
| | *left* | 6.48 ± 1.03 | 7.33 ± 0.58 | **0.002*** | 7.14 ± 0.91 | 6.67 ± 1.24 | 0.052 |
| *III carpometacarpal joint* | *right* | 6.48 ± 1.08 | 6.95 ± 0.92 | 0.195 | 6.86 ± 1.11 | 6.67 ± 1.20 | 0.360 |
| | *left* | 6.95 ± 0.74 | 7.14 ± 0.57 | 0.377 | 6.76 ± 1.04 | 6.52 ± 1.17 | 0.353 |
| *V carpometacarpal joint* | *right* | 6.52 ± 0.75 | 7.05 ± 0.67 | **0.018*** | 6.81 ± 1.29 | 6.71 ± 1.31 | 0.971 |
| | *left* | 6.48 ± 1.81 | 7.14 ± 0.85 | 0.140 | 6.90 ± 1.04 | 6.71 ± 1.19 | 0.475 |
| ***PROs*** | | | | | | | |
| *Fact-GOG-ntx* | *Score* | 28.38 ± 7.68 | 33.10 ± 8.51 | **0.017*** | 34.24 ± 5.58 | 33.14 ± 7.23 | 0.461 |
| *NR Scale* | *Numbness and Tingling* | 4.67 ± 2.01 | 2.00 ± 2.07 | **0.001**** | 3.34 ± 1.11 | 2.52 ± 1.69 | 0.060 |
| | *Pain* | 1.76 ± 3.02 | 0.52 ± 1.54 | 0.092 | 0.52 ± 1.44 | 0.42 ± 9.87 | 0.781 |
| *EORTC-QLQ-C30* | *Physical Functioning* | 68.6 ± 22.2 | 70.1 ± 24.3 | 0.704 | 80.6 ± 14.7 | 85.7 ± 26.0 | 0.689 |
| | *Pain* | 37.1 ± 30.3 | 32.5 ± 32.3 | 0.490 | 30.8 ± 26.8 | 34.1 ± 32.3 | 0.694 |
| | *Global Health State* | 33.9 ± 22.5 | 55.2 ± 23.3 | **0.001**** | 45.3 ± 26.9 | 50.0 ± 22.9 | 0.518 |
